# A randomized trial on differential changes in thought and affect after mindfulness versus dyadic practice indicates phenomenological fingerprints of app-based interventions

**DOI:** 10.1038/s41598-023-40636-1

**Published:** 2023-08-24

**Authors:** Paul Petzold, Sarita Silveira, Malvika Godara, Hannah Matthaeus, Tania Singer

**Affiliations:** Social Neuroscience Lab, Max Planck Society, Bertha-Benz-Str. 3, 10557 Berlin, Germany

**Keywords:** Psychology, Clinical trial design

## Abstract

Contemplative practice has demonstrated benefits for mental health and well-being. Most previous studies, however, implemented in-person trainings containing a mix of different, mostly solitary, practices and focused on pre- to post-training outcomes. In this randomized trial, we explore the immediate differential efficacy of two daily app-delivered practices in shifting emotional (valence, arousal) and thinking patterns (thought content on future-past, self-other, positive–negative dimensions). For 10 weeks of daily training, 212 participants (18–65 years) performed either a novel 12-min partner-based socio-emotional practice (Affect Dyad) or a 12-min attention-focused solitary mindfulness-based practice. Using ordinal Bayesian multilevel modeling, we found that both practice types led to more positive affect and higher arousal. However, whereas mindfulness-based practice partly led to a decrease in active thoughts, particularly in future-, other-related and negative thoughts, the Dyad in contrast led to increases in other-related, and positive thoughts. This shift towards more social and positive thoughts may specifically support overcoming ruminative thinking patterns associated with self-related and negative thought content. Overall, these differential findings may help inform the adaptation of scalable app-based mental trainings in different segments of the population with the goal to improve mental health and well-being.

## Introduction

In an interconnected globalized world with the ubiquitous availability of smartphones and wireless internet, online applications to improve mental health and psychological well-being are flourishing. One specific type of such intervention that has gained much attention in recent years is secular meditation practice constituting low-dose or fractional versions of prevailing mindfulness or compassion-based mental training programs^1,2^. Presumably accelerated by the worldwide COVID-19 pandemic, the number of meditation app users has tripled since 2019 to an outstanding number of 185 million users and is expected to grow by additional 100 million users within the next five years^3^. These numbers highlight the potential of app-delivered mental trainings as a far-reaching and scalable intervention tool. Despite an increase in scientific evidence of the multiple benefits of contemplative practice on mental health and well-being^4^, the majority of research in this field is still based on in-person trainings, and a mixture of classic solitary meditation practices^5^. In contrast, we here provide a direct systematic comparison of two different types of mental practices, both performed over 10 weeks using a smartphone app, in their efficacy to shift thought patterns and emotional well-being in direct response to daily practice. Moreover, we investigate the specific effect of a rather novel partner-based practice, the so-called Affect Dyad^6^ in contrast to classic solitary mindfulness practice on changes in this phenomenological space.

Rooted in contemplative traditions from Eastern cultures, a large amount of secularized mental training programs were developed over the last decades, many of which can be categorized as mindfulness or compassion-focused contemplative trainings. Most of the mindfulness-based programs are derivates of Mindfulness-Based Stress Reduction (MBSR;^2^) and Mindfulness-Based Cognitive Therapy programs (MBCT;^7^). Mindfulness-based approaches aim at fostering present-moment awareness with a non-judgmental attitude, oftentimes by means of attentional focus on internal and external sensations^5^. Compassion-based interventions on the other hand, such as the Mindful Self-Compassion program (MSC;^1^), Compassion Cultivation Training program (CCT;^8^), and Compassion-Focused Therapy (CFT;^9^) aim at enhancing socio-affective skills, including compassion towards self and others, loving-kindness, prosocial motivation, altruism and empathy^4^. Contemplative practice has repeatedly been shown to yield positive effects in several domains, leading to a multitude of meta-analyses on a variety of health-related outcomes. Mindfulness-based interventions have been shown to reduce stress and symptoms of anxiety or depression, improve mental health-related quality of life^10–12^, sleep quality, and insomnia^13,14^, and may also help alleviate (chronic) pain^15,16^. Similarly, loving-kindness and compassion-based programs were found to reduce symptoms of depression, anxiety, and psychological distress while increasing positive emotions, life satisfaction, mindfulness, and (self-) compassion^17–21^. Whereas most of these traditional secular mindfulness- or compassion-based programs are of 8 weeks duration and contain a mix of different types of mental practices, a previous 9-month longitudinal project designed by one of the authors, the ReSource project^22^ compared three 3-month training modules with distinct practice focus, that is attention-focused, socio-emotional or socio-cognitive practice types. Comparative findings indeed highlight the domain-specific effects of different modules on diverse outcomes^4^. It was found that while three months of attention-based mindfulness training improved attention and working memory^23,24^, compassion- and care-based socio-emotional mental practices were particularly effective in increasing compassion and altruistic prosocial behaviors^24,25^. Differential effects of type of practice were also observed for changes in brain structure and function, leading to increases in cortical thickness in different brain networks supporting attention, compassion, and cognitive perspective-taking^26^. Despite evidence for the relevance of the type of practice, systematic investigation of differential practice effects is still scarce, especially in the domain of app-based interventions. To fill this gap, the current study seeks to systematically compare the effects of two low-dose app-delivered practice types with either attention-based mindfulness or socio-emotional training focus.

Apart from differences in training targets and content, contemplative practices further differ with regard to training formats. As such, they can be conducted in the form of solitary meditation, which is the case for most mindfulness programs. However, more recently, contemplative practices have also been performed in interpersonal settings, including group settings or partner-based exercises such as Insight dialogues^27^, inquiry methods^28^, or interpersonal retreat formats^29^. In the ReSource project, a specific partner-based practice, the so-called Contemplative Dyad, has been formally introduced as daily practice with weekly changing partners performed over several weeks^6^ and found to be particularly beneficial for fostering social connectedness. A Dyad can be conceived as a meditation practice together with a partner: one partner is listening empathically without interrupting, while the other partner is elaborating on a question. After about 6 min partners switch roles. While dyadic practices can focus on different content, based on notions of a motivational care-system^30^, the “Affect Dyad” that was implemented in the present study aimed to cultivate socio-emotional qualities such as acceptance of difficult emotions, gratitude, and care. The two partners take turns in exploring the effects of negative emotions or gratitude experienced during the past day, and the bodily sensations associated with these emotions. In the ReSource project, only the socio-cognitive and socio-emotional modules, which among other practices included partner-based dyadic exercises, were able to reliably reduce cortisol stress reactivity after a social stressor^31^. Overall, findings suggest that the format of mental practices leads to different outcomes on the level of physiology, biology, subjective experience, and behaviors ^4^. Given the relative novelty of such dyadic mental practices, the current study focused on directly comparing the effects of daily solitary mindfulness-based practice with daily socio-emotional intersubjective practice in the form of the Affect Dyad, both conducted using a mobile app.

Furthermore, while the majority of contemplative training studies have focused on measuring training-related effects from before to after several weeks or months of training on outcomes such as neurophysiological, psychological, and other health-related markers^10–21,32^, some contemporary studies have explored the immediate effects of specific practices in the subjectively experienced phenomenological space. Accordingly, it has been found that alterations of phenomenological perceptions of body, space, and time can be instantly evoked by meditation practice^6,33–36^. In the context of the above-mentioned ReSource project, micro-phenomenological qualitative interviews about the subjective experience of different types of practices (i.e., loving-kindness meditation, breathing meditation, and observing thought meditation) revealed distinct patterns of emotional tone, semantic space, body space or perceived colors in immediate response to each practice^37^.

A core aspect of phenomenological content is self-generated thought, that is, mental content and thinking patterns that are independent of the external environment or sensory stimuli^38^. Importantly, given a temporal connection between self-generated thought and future mood, the phenomenological content of self-generated thought can have long-term impacts on mental health and well-being^39–43^. In line with this, individuals with major depressive disorder (MDD), borderline personality disorder (BPD), or narcissistic personality disorder (NPD) demonstrate specific patterns of socio-temporal content of self-generated thought, including more negative, self- and past-oriented thoughts in MDD^44^, more positive, self- and future-oriented thoughts in NPD^45^, and more negative thoughts with higher variability in self- and other-orientation in BPD^46^. The impact of mindfulness-based practice on thought content relates to its focus on non-judgmental present-moment awareness. Previous evidence from the ReSource project investigating changes in both thought content as well as affective tone and arousal indicates a general decrease in self-generated thought content (i.e., future-, past-, other-related, positive, and negative thoughts) through mindfulness training and an increase in positive and other-related thoughts in the compassion-based training^35^. These changes in thought patterns have been ascribed to distinct phenomenological fingerprints of contemplative practices, which are characterized by specific patterns of mental states. As such, the observed decreases in thought content have been related to a mindfulness practice-induced fingerprint of an overall “calming of the mind”. The fingerprint induced by compassion-based practice on the other hand was characteristic of a shift of subjective experiences towards care, gratefulness, and compassion^35^. Specifically, loving-kindness meditation invoked positive thoughts that clustered with thoughts about others. While in the case of loving-kindness meditation explicitly involves the extension of love and good wishes to close people in one’s life^47^, it remains an open question whether more implicit types of compassion practice can have a similar effect.

Besides thought content, also phenomenology of affect and affect dynamics are crucial to mental health and psychopathology^48,49^. In fact, affect levels have been shown to predict treatment outcomes as well, making them a crucial neurocognitive marker of psychopathology^50^. In the ReSource project, all types of meditation led to an increase in positive affect valence and arousal^35^, while other studies found both positive and negative affective valence to be reduced by mindfulness-based practice^51^. These results speak for the potential of different types of practice to differentially alter thought content and patterns as well as emotional well-being. Unfortunately, however, the ReSource project did not involve a phenomenological analysis of the specific effect of the rather novel Affect Dyad, which given its partner-based format and its focus on socio-affective experiences and body sensations can be expected to induce specific and differentiable shifts in emotional well-being and thinking patterns as compared to more classical solitary mindfulness meditations.

Here, we directly compare the specific shifts in thought content as well as emotions evoked by daily 12-min app-delivered dyadic practice versus classic solitary mindfulness practice using the so-called “Cube of Thought” that assesses three dimensions of thought content, that is (1) temporality (past—future), (2) social orientation (self—other), and valence of thoughts (positive—negative) ^43^, and the so-called “Affect Grid” with separate dimensions for affect valence and arousal, here defined as the physiological level of stimulation and energy^52^. More specifically and given the mechanistic relevance of self-generated thought patterns and affective states for mental health, this study aims to extend previous research on distinct phenomenological fingerprints of different types of contemplative trainings^35^ to the systematic investigation of the common and differential phenomenological state changes elicited by daily practice of app-delivered attention-focused mindfulness-based practice as compared to the partner-based Affect Dyad. Based on theoretical notions of a motivational care system^30^, and previous findings on compassion-based practice^6,35^, we thereby expected the socio-affective Affect Dyad to specifically instill care and compassion, and thus positive other-related thoughts and positive affect valence, and the mindfulness-based training to generally reduce thought content on all dimensions as indicators of a calmer, “emptier” mind. While the development of “selflessness” is a general pursuit of contemplative practice, previous evidence does not point to a reduction of self-related thought through practice. We will therefore explore the malleability of self-related thoughts by socio-emotional and mindfulness practices in our sample. With regards to affective states, we expected both trainings to increase positive affect, yet aimed to explore differential practice effects on affect valence and arousal. Furthermore, it will be explored whether such brief 12-min daily app-delivered mental trainings lead to lasting effects on socio-temporal thought patterns and mood over the duration of the 10 weeks training.

## Methods

### Participants

The current study is part of the second phase of the larger CovSocial project, a two-phase study investigating the psychosocial impact of the COVID-19 pandemic in a large community sample in Berlin, Germany. The first phase sought to evaluate longitudinal changes in vulnerability, resilience, and social cohesion during the pandemic (January 2020 to April 2021;^53^), whereas the subsequent second phase (August 2021 to February 2022) studied the effectiveness of 10-week app-delivered socio-emotional and mindfulness-based interventions in reducing stress, loneliness, and anxiety, and in enhancing resilience, empathy, and compassion^54^. All participants with completed datasets of retrospective longitudinal assessments in phase 1 (*n* = 3522) were invited for pre-screening of the phase 2 intervention study. Inclusion criteria involved age between 18 and 65 years, German language proficiency, and registered residency in the city of Berlin. Exclusion criteria for phase 2 included no internet access or necessary technical equipment, educational background in psychology, regular spiritual practice, medication that influences physiological markers, previous participation in stress reduction programs, chronic illness or pain, a current psychiatric diagnosis received in the past two years as declared in self-report, or scores above cut-off on clinical scales such as the Toronto Alexithymia Scale-20 (TAS-20 > 60;^55^), Patient Health Questionnaire-9 (PHQ-9 > 19;^56^), and Generalized Anxiety Disorder-7 (GAD-7 > 15;^57^).

**Figure 1:**
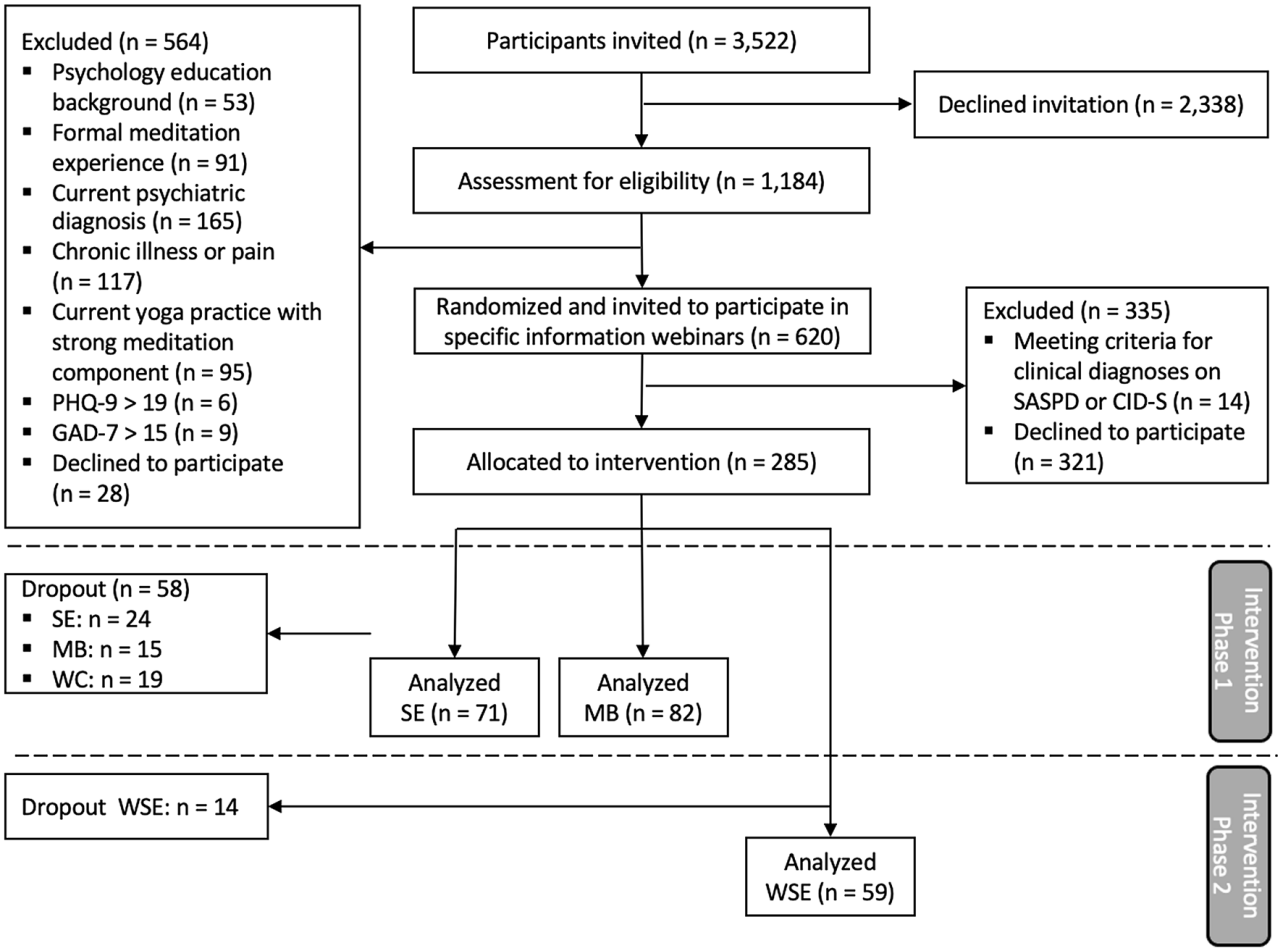
Participant flow diagram. SE/WSE = (waitlist) socio-emotional training, MB = mindfulness-based training, WC = waitlist control group.

Based on a-priori power analysis for CovSocial phase 2 (Supplement 1), we aimed to recruit 100 participants per intervention group. After exclusion conditions were met, 620 subjects were randomly assigned by a senior researcher in the project to one of three groups: socio-emotional mental training (SE), mindfulness-based mental training (MB), and waitlist control (WC/WSE), using computer-generated numbers in a block randomization technique with 1:1:1 allocation. Participants were invited to information webinars of 90 min that served as a general introduction to the theoretical background of contemplative mental training programs. After these webinars, 298 individuals provided written informed consent. Certified meditation teachers conducted screening calls, using the Standardized Assessment of Severity of Personality Disorder (SASPD;^58^) and Composite International Diagnostic Screener (CID-S;^59^) to exclude participants with above cut-off levels. The resulting *n* = 285 phase 2 sample started with 95 participants in the SE group, 97 participants in the MB group, and 93 participants in the WC group, of whom 59 chose to continue with the socio-emotional Dyad intervention (WSE) after the first posttest. Since the current study reports on data that was assessed in context of the daily mental practice, only SE, MB, and WSE groups are included in statistical analyses. Due to participant dropout before and during the intervention phase, analyses are based on 71 participants in the SE intervention, 82 participants in the MB intervention, and 59 participants in the WSE intervention (for sample demographics see Table 1; for details about participant exclusion, and dropout, see Fig. 1).

**Table 1:** Sample demographics split by intervention group.

Characteristic	SE n = 71	MB n = 82	WSE n = 59
Age in years, mean (SD)	43.34 (11.96)	43.48 (11.56)	44.63 (10.91)
Females, *n* (%)	55 (77.5%)	57 (69.5%)	43 (72.9%)
Background of migration, *n* (%)	4 (5.6%)	10 (12.2%)	3 (5.1%)
Years of education, mean (SD)	18.34 (4.12)	17.05 (3.41)	18.32 (2.89)
Married or cohabiting, *n* (%)	25 (35.2%)	28 (34.1%)	21 (35.6%)
Lifetime prevalence of psychiatric disorder, *n* (%)	16 (22.5%)	14 (17.1%)	12 (20.3%)
Net income > Berlin average^1^, *n* (%)	45 (63.4%)	56 (68.3%)	41 (69.5%)
Employed full-time, *n* (%)	35 (49.3%)	53 (64.6%)	37 (62.7%)

*SE/WSE* (waitlist) socio-emotional intervention, *MB* mindfulness-based intervention.

^1^The average monthly net income in Berlin is approximately 2175€ (Amt für Statistik 2019; https://www.statistik-berlin-brandenburg.de/publikationen).

All participants provided written informed consent prior to their participation. All methods and procedures contributing to this work were in accordance with the ethical standards of the relevant national and institutional committees on human experimentation and with the Helsinki Declaration. The study protocol was approved by the ethics commission of the Charité—Universitätsmedizin Berlin (#EA4/081/21). The study was registered with ClinicalTrials.gov on (17/05/2020, #NCT04889508).

### Procedure

In this randomized trial with two parallel intervention arms, we compared two distinct app-delivered 10-week online interventions, a socio-emotional training with daily Affect Dyad practice and an attention-focused mindfulness-based mental training with daily guided meditation. In addition to the parallel trial design, the waitlist control group underwent socio-emotional training after posttest 1. Interventions and study measures were delivered through a non-commercial smartphone app that was developed as a research tool for the ReSource project^22^ and was adapted for the CovSocial project (see Supplement 2). Introduction to installation, default settings, and functions of the CovSocial app were given to SE and MB participants at the study pretest, and to WSE participants at posttest 1. Prior to the intervention period, all participants received 2.5 h of formal introduction to contemplative trainings, and two 2.5 h long group-specific onboarding webinars including the theoretical and practical introduction to respective training programs (see Supplement 3).

In both mental training programs, daily app-delivered practice was conducted six days per week and complemented by weekly two hours of online coaching sessions conducted by trained meditation teachers on the seventh day. Assignment to one of the four teachers was depending on the participants’ availability, yet the teacher and time of coaching were counterbalanced between the intervention groups. Each teacher was assigned a group of approximately 14 to 24 participants over the 10 weeks. Each coaching session aimed to deepen the participants’ understanding of different aspects of their respective practice and to help integrate it in their life. The topics of coaching sessions in the socio-emotional training program revolved around social connectedness, empathic non-judgmental listening, interoceptive body awareness, acceptance of difficult emotions or stress, and the cultivation of care and gratitude. The topics of coaching sessions in the mindfulness-based training program included basics of breathing meditation, body awareness, sensory perception and awareness, and dealing with difficult emotions, in line with the aim of the training to foster present-moment attention, interoceptive body awareness, and an attitude of dignity and receptivity towards the self and the body. A detailed description of the teacher training and coaching protocol can be found in Supplement 3.

### Socio-emotional training

The daily practice of the socio-emotional training program was the Affect Dyad, a 12-min partner-based exercise, in which two randomly matched participants take on the roles of a speaker and a listener. Thereby, daily appointments could be scheduled in the app by both partners, and participants were reminded of the appointment 10 min before the practice via a push notification. The speaker explores two questions posed by the listener. The first question is: “Please tell me about a situation of your last 24 h, in which you experienced a difficult emotion, and how it felt in your body.” After elaborating for 2.5 min, the listener is instructed to say: “Thank you for sharing. Now, please tell me about a situation of your last 24 h, in which you felt grateful and how that felt in your body.” After one minute of silence, the roles are switched, and the procedure is repeated. The Dyad starts and ends with a period of silence to drop everything shared and heard. The listener is instructed to listen non-judgmentally and empathically. The partner-based practice aims to foster positive qualities and social capacities such as empathy, acceptance of self and others, gratitude, acceptance of difficult emotions, and social connectedness.

### Mindfulness-based training

The mindfulness-based mental training program included daily attention-based mindfulness practices that were previously implemented in the Presence module of the ReSource project (for details, see^22^), and included classic breathing meditation, attention-based mindfulness on sounds, and open presence meditation. Daily practice of the meditation was guided by pre-recorded audio files in the app. In daily practices, participants were instructed to monitor and direct the focus of their attention to their breath, auditory stimuli, or internal and external sensations (depending upon the meditation type). In general, these mindfulness-based practices aim at cultivating non-judgmental awareness of the present moment and interoceptive body awareness.

### Measures

**Figure 2:**
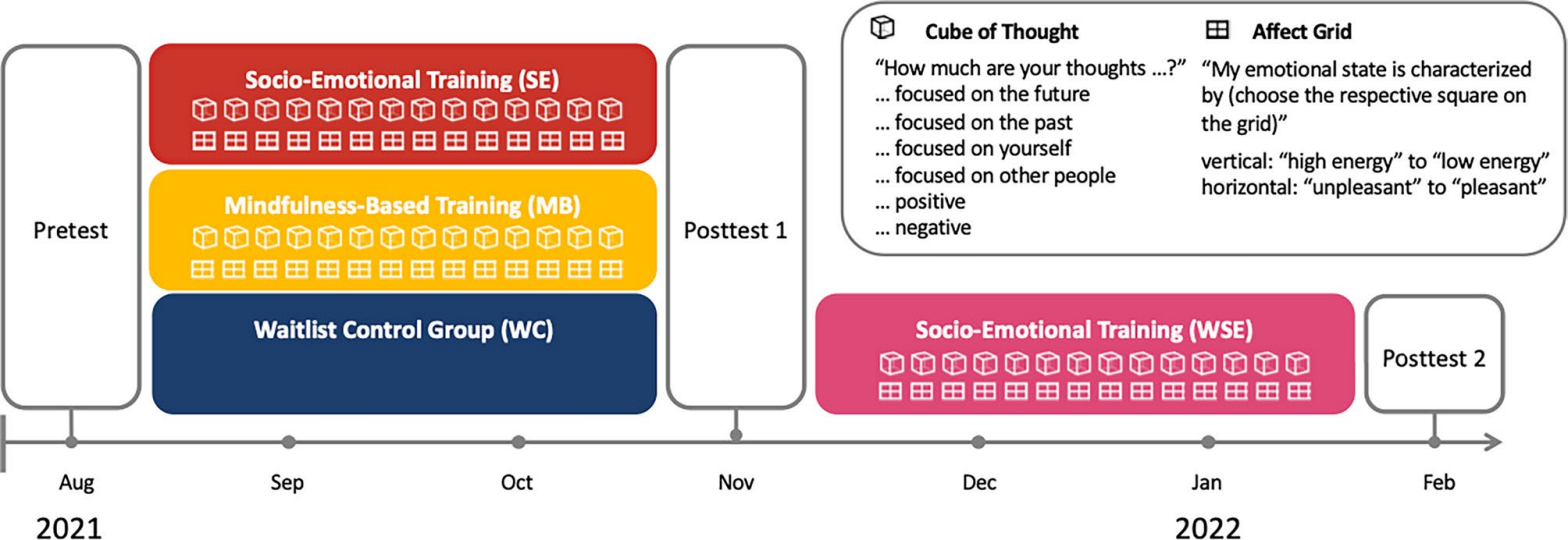
CovSocial Phase 2 study design with assessments of thought content and affect before and after daily practice in the mental training conditions during two intervention phases.

The CovSocial project involved a wide range of assessments, including measures of stress, mental health, and psychological resilience as well as epigenetic markers and biological markers of inflammation (for a full list of measures, see^54^). In the present study, we focused on practice-induced phenomenology of thought content measured with the Cube of Thought^43^ and of affective state measured with the Affect Grid^52^. The Cube of Thought is a self-report measure of self-generated thoughts. It consists of three bipolar dimensions, each assessed with two items: temporality (past- or future-oriented thoughts), social orientation (self- or other-related thoughts), and valence (positive or negative thoughts). Participants were asked to evaluate their current thoughts on a Likert scale from 0 (“not at all”) to 4 (“very much”). The Affect Grid assesses affective states on two dimensions for affect valence and arousal, with ratings ranging from -4 (“unpleasant”) to 4 (“pleasant”) for affect valence and from -4 (“low energy”) to 4 (“high energy”) for affective arousal. Further, participants’ motivation to perform an upcoming mental practice was assessed right before each daily practice in a single item on a rating scale from 0 (“not at all”) to 8 (“very much”). All outcome variables were measured using the CovSocial smartphone app directly before and after daily practice (Fig. 2). Within the app, pre-practice items were displayed before recordings of mindfulness practice could be accessed or dyadic partners were connected. Similarly, post-practice items were presented in direct succession to the practice before the app was closed. To reduce the time cost of app use with direct relation to daily practice, Affect Grid and Cube of Thought were not presented every day, but on three days of the week in a pseudo-random order with other study measures that are not reported here. In total, participants who followed the instructions to complete the mental practice 6 days a week would have completed each question 60 times (i.e., twice on 30 days) in the 10-week intervention. Compliance with practice was assessed as the percentage between the total count of performed daily practice during the intervention period and the highest possible number of daily practices. Overall, compliance rates were high in all three intervention groups, with the socio-emotional trainings having considerably lower variances (SE: Mean = 87.9, SD = 9.05; MB: Mean = 82.3, SD = 18.5; WSE: Mean = 89.5, SD = 7.82). Taken together, we recorded 19,710 data points for all participants across all outcome variables.

### Data analysis

To investigate changes in the Cube of Thought and Affect Grid across time and groups, ordinal Bayesian hierarchical modeling using a probit function was conducted to account for both the ordinal and hierarchical structure of the data (for an introduction on ordinal Bayesian modeling, see^60^). The ordinal models assume a latent continuous and normally distributed variable from which the observed single-item Likert scores originate. To test for within- and between-person training effects, a two-level hierarchical linear model was fitted for each outcome variable using the brms package^61^ in the open-source software R Version 4.2.0^62^. Data was structured into measurement days (level 1) nested within persons (level 2). Each model included fixed effects for the type of intervention (a categorical variable with three levels; *intervention*), whether the measurement was taken before or after the intervention (a dummy coded variable with pre-practice scores as reference condition; *occasion*), and measurement occasion (a continuous variable; *time*), as well as their respective two- and three-way interactions. Contrasts for intervention groups were dummy-coded with the mindfulness-based mental training condition defined as the reference group. For each outcome variable, a two step-approach was applied. In a first step, the best-fitting model was identified using weakly informative priors, 1000 burnins, 4000 iterations, and four chains. We compared a linear model, random intercept model, two random slope models with random effects for *occasion* and *occasion x time* interaction respectively using leave-one-out cross-validation^63^. In a second step, best fitting models of the first step were used for sensitivity analyses of each outcome variable to check for prior sensitivity using weakly and super weakly informative priors. Included covariates were participants’ *age*, *sex*, *motivation*, and *weekend* (a dichotomous variable indicating whether the training took place on a weekend). Metric variables were grand mean centered and scaled in all models. Hypothesis testing on estimated parameters was performed using the region of practical equivalence (ROPE) and the highest density intervals (HDI) of posterior distributions^64^. In this regard, an HDI outside the ROPE is indicative of a substantial effect. Detailed information on all models is available in the supplementary material (Supplement 4) and on Open Science Framework (https://osf.io/vcasn/). Consistent across all outcome variables, models with random effects for *occasion* and *time* showed the best model fit in comparison to other tested models using leave-one-out cross-validation. Since parameter values weren’t sensitive to the tested prior distributions, results are reported for models using weakly informative priors (for further information regarding model selection and parameter estimation, see Supplement Tables S1, S2, and S3, Figs. S1 and S2).

## Results

**Figure 3:**
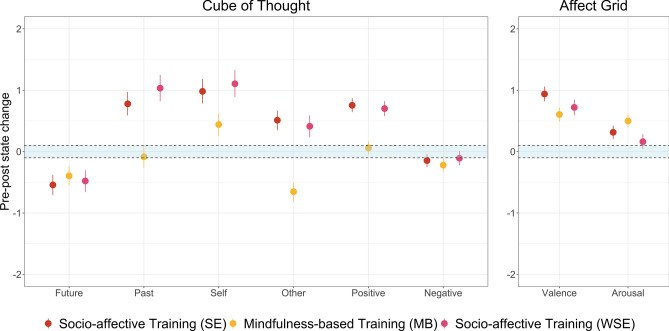
Phenomenological state changes from before to after daily practice. Estimates and 95% credibility intervals for model-derived state changes of the Cube of Thought and Affect Grid in the three mental training groups. The blue marked area depicts the region of practical equivalence (ROPE).

In the following we report the results of the ordinal Bayesian mixed models for each outcome variable for practice-induced pre-post state changes, starting with dimensions of the Cube of Thought (temporality, social orientation and valence of thoughts), followed by the Affect Grid. Subsequently, we report results for changes over time. Figure 3 depicts the ordinal Bayesian mixed model-derived pre-post state changes in thought content and emotional well-being and their 95% credibility intervals on the latent continuous variable. Model estimates of main effects are presented in Table 2.

**Table 2:** Model estimates of immediate practice-induced main effects in all intervention groups.

	SE		MB		WSE	
	*b*	95% CI	*b*	95% CI	*b*	95% CI
CoT—Future	−0.54	[−0.71, −0.38]	−0.39	[−0.55, −0.24]	−0.48	[−0.66, −0.3]
CoT—Past	0.78	[0.59, 0.97]	−0.08	[−0.26, 0.1]	1.03	[0.82, 1.25]
CoT—Self	0.98	[0.78, 1.18]	0.44	[0.26, 0.62]	1.11	[0.89, 1.33]
CoT—Past	0.51	[0.35, 0.67]	−0.65	[−0.8, −0.5]	0.41	[0.23, 0.59]
CoT—Positive	0.75	[0.64, 0.87]	0.06	[−0.05, 0.17]	0.70	[0.58, 0.82]
CoT—Negative	−0.15	[−0.25, −0.04]	−0.22	[−0.32, −0.12]	−0.11	[−0.22, 0.01]
AG—Valence	0.94	[0.82, 1.06]	0.60	[0.49, 0.72]	0.72	[0.59, 0.85]
AG—Arousal	0.31	[0.21, 0.43]	0.50	[0.39, 0.61]	0.16	[0.04, 0.28]

*b* = mean regression coefficient across persons, *95% CI* 95% credibility interval. *SE/WSE* (waitlist) socio-emotional training, *MB* mindfulness-based training, *CoT* cube of thought, *AG* affect grid.

### Cube of thoughts

With regards to temporality of thoughts, both MB and socio-emotional (SE, WSE) practice led to substantial immediate decreases in future-related thoughts when comparing post-practice and pre-practice scores. Neither SE nor WSE substantially differed from MB. Past-related thoughts remained stable in the MB, but increased in both SE and WSE training groups from pre- to post-practice, with changes being substantially higher in both groups compared to MB (SE: *b* = 0.86, 95% CI = [0.6, 1.13], WSE: *b* = 1.12, 95% CI = [0.84, 1.39]).

With regards to social orientation of thoughts, in both mental trainings, increased self-related thoughts were observed from before to after the daily practice. Both SE and WSE had stronger increases in self-related thoughts compared to MB (SE: *b* = 0.54, 95% CI = [0.27, 0.81]; WSE: *b* = 0.66, 95% CI = [0.38, 0.95]). For other-related thoughts, a considerable practice-related decrease was found for MB. Conversely, other-related thoughts increased in both SE and WSE training groups. The differences in pre- to post-practice changes between both socio-emotional training groups and the MB training group were highly substantial (SE: *b* = 1.16, 95% CI = [0.94, 1.39]; WSE: *b* = 1.07, 95% CI = [0.83, 1.3]).

Whereas in the MB group no practice-related changes were found in positively valenced thoughts, both socio-emotional training groups (SE, WSE) showed highly substantial increases. These increases in positive thoughts were substantially higher compared to MB (SE: *b* = 0.69, 95% CI = [0.54, 0.85]; WSE: *b* = 0.64, 95% CI = [0.48, 0.8]). Only MB had a (small) substantial decrease in negative thoughts, whereas the HDIs of SE and WSE partially overlapped the region of practical equivalence. There were no meaningful differential effects of practice on negative thoughts between SE or WSE and MB groups (SE: *b* = 0.07, 95% CI = [−0.07, 0.22], WSE: *b* = −0.11, 95%-CI = [−0.04, 0.27]).

### Affect valence and arousal

All three intervention groups showed substantial increases in positive affect valence post-practice compared to pre-practice. These increases were considerably higher in SE (*b* = 0.33, 95% CI = [0.17, 0.5]), but not WSE (*b* = 0.12, 95% CI = [−0.05, 0.29]) compared to MB. Similarly, all intervention groups showed practice-related increases in affective arousal, although only of substantial magnitude for MB and SE, since the HDI of WSE partially included the ROPE. Yet these increases were only substantially lower in WSE compared to the MB group (SE: *b* = −0.18, 95% CI = [−0.34, −0.03]; WSE: *b* = −0.34, 95% CI = [−0.5, −0.18]).

### Practice-related changes over 10-weeks of intervention

Longitudinal changes in pre-practice thoughts (Cube of Thought) and affect (Affect Grid) over the course of the 10 weeks of intervention were reflected in parameter estimates of the two-way interactions between *intervention* group and *time* (for longitudinal pre-practice affect and thought) or three-way interactions between *intervention*, measurement *occasion* and *time* (for longitudinal trajectories of pre- to post-practice changes). Although small changes could be observed over the course of the 10-week intervention period, all highest density intervals fell at least partially within the ROPE, and thus indicating no conclusive evidence for lasting changes over time.

Additionally, we explored if pre- to post-practice changes evolved over time as reflected in three-way interactions between the variables *intervention*, *time*, and *occasion*. Here, we found substantial changes in self-related thoughts: Whereas the pre- to post-practice difference in MB decreased over time (*b* = −0.21, 95% CI = [−0.29, −0.14]), both SE and WSE groups showed increases (SE: *b* = 0.27, 95% CI = [0.13, 0.42]; WSE: *b* = 0.17, 95% CI = [0.02, 0.32]). Yet only the highest density interval of MB and SE fell completely outside the ROPE indicating evidence for substantial change. The highest density interval of WSE contained at least partially the ROPE and thus we refrain from making claims about changes over time in these variables.

## Discussion

The primary aims of this study were to investigate differential immediate effects and longitudinal effects over 10 weeks of two app-delivered daily practices on emotional well-being and thinking patterns. Specifically, a goal of the current study was to directly compare a novel partner-based socio-emotional mental practice, the Affect Dyad^6^, with more widely studied attention-focused mindfulness-based mental practices^65^, in its effects on different dimensions of thought content as measured through the “Cube of Thought”^43^ and affect valence and arousal as measured with the “Affect Grid”^52^ in the context of the second phase of a Berlin-based longitudinal mental health study, the CovSocial project^54^. It was expected that mindfulness-based practice can reduce the intensity of active thoughts, while the socio-emotional Affect Dyad practice was expected to instill more social and positively valenced thoughts.

Ordinal Bayesian mixed modeling revealed both similarities as well as considerable differences between socio-emotional dyadic and mindfulness-based mental practices on a phenomenological level. With regards to thought patterns, we found the daily practice of the Affect Dyad in itself as well as in comparison to the mindfulness-based practice indeed to be effective in increasing positive and other-related thoughts. Beyond these expected effects, the dyadic practice also led to a distinct increase in self- and past-related thoughts. After mindfulness-based practice, on the other hand, expected decreases in future-, other-related and negative thoughts could be observed, while past-related and positive thoughts remained unchanged, and self-related thoughts increased. Both practice types led to increases in positive affect, which was higher after dyadic practice in the SE group. Affect arousal increased in MB and SE groups. Taken together, we found evidence for commonalities in the immediate effects of both types of daily app-based practices, speaking to their efficacy in boosting positive affect. But more importantly, we also observed differential phenomenological fingerprints in evoked patterns of thought and feeling states between both practice types. Whereas mindfulness training seemed to be more efficient in instantly reducing thought content on several dimensions, the partner-based Dyad seemed to instantly instill more positive thoughts as well as thoughts about other people.

Based on the findings of Kok and Singer^35^, the observed fingerprint for the mindfulness-based training may be related to what has been broadly described as “calming of the mind”, characterized by a general decrease in thought content. Specifically, we found decreases in future-, other-related, and negative thoughts in direct response to mindfulness-based practice, while past-related and positive thoughts showed no change. Deviations from previous findings might be associated with the specific content of mindfulness practice. In our study, using practices that first focused on breathing meditation and body scan, and successively on sensory awareness and open awareness (see intervention protocol Supplement 3), self-related thoughts on average increased immediately after daily mindfulness practice. This increase in self-related thoughts might relate to the degree of self-regulation and thus self-awareness that has been associated with mindfulness practice^66^. The increase in self-related thought was, however, substantially lower than after partner-based socio-emotional practice.

While previous findings on mindfulness practice-related changes in affect valence have been inconsistent, with some suggesting a calming decrease in both positive and negative affect^51^, and others suggesting an increase in positive affect and arousal^35^, our findings replicate the latter effects on more positive valence and higher arousal after compared to before mindfulness practice. Thus, while a calming effect was observed for several aspects of thought content, the affective state seemed to be impacted in different ways, hinting to the positive effects of mindfulness practices on mood and energy levels.

Our findings on the specific effects of the Affect Dyad are partly in line with previous findings on specific phenomenological fingerprints of socio-affective meditation practices observed in the compassion-based Affect module of the 9-month ReSource project^22^. More specifically, Kok & Singer^35^ observed that the daily practice of loving-kindness meditation specifically changed the valence of thought content, such that thoughts became more positive and less negative. Besides, the positively valenced thoughts clustered with thoughts about others and the self, which had been ascribed to the training focus of loving-kindness meditation on good wishes to other people^47^. Our findings provide evidence that the brief app-based Affect Dyad has similar effects on increased positive thoughts, and increased thoughts about others, yet no changes in negative thoughts were found. Increases in positive and other-related thoughts were distinctly stronger than after mindfulness practice. The additional increase in thinking about oneself and the past can be seen as a direct validation of the dyadic mental exercise itself, in the sense that the Affect Dyad requires individuals to recall and elaborate on two situations that they experienced in the last 24 h. Thus, the contemplative part required participants to think of the past to be able to recall affective and bodily states experienced in those moments, that is the practice generally requires heightened past- and self-related thinking. In this sense, the finding of relatively unaffected negative thought content despite explicit elaboration on a situation that evoked difficult emotions, might be seen as an acceptance of challenging situations without focusing on the negative experience itself. Interestingly, however, the increase in other-related thoughts can be seen as validation that dyadic partners were indeed also empathically focusing on the experiences of others in their every day life. It has been suggested that such partner-based mental practices can activate a motivational care-system, which promotes social connection and feelings of nurture, compassion, and acceptance^4,30^. Together with the practice-related elaboration of feelings of gratitude, which oftentimes involves positive thoughts on others, as well as the practice of non-judgmental and empathic listening, a shift of focus on positive thoughts and thoughts about others thus highlights the immediate specific effect of the Affect Dyad on this social aspect of thought content. With regards to emotions, daily practice again led to more positive affect and higher arousal in both training groups (SE and WSE), and this increase in positive affect was even higher than after mindfulness practice. In contrast to the mind-calming effects of classic mindfulness practices, it seems that the socio-emotional dyadic exercises foster positive social emotions as well as positive other-related thinking patterns more strongly.

While on average direct effects of daily practice could be observed, a confounding effect of the weekly coaching session cannot be excluded. Coaching sessions were standardized and kept as consistent as possible between the two interventions. However, they were characterized by differences related to practice-specific focus. Particularly the longitudinal decrease in the direct effect of mindfulness-based practices on self-related thoughts over the course of the 10-week intervention period might be related to weekly coaching sessions. Thereby, particularly at the beginning of the intervention period, the focus of coaching sessions revolved around internal sensations such as breathing, body awareness and emotions (see coaching protocol in Supplement 3). Subsequent coaching sessions focused on external sensory experiences (e.g., hearing and sensory awareness) and open awareness, which likely activated less self-reference. While we abstained from including time-lagged models of change in the current analyses, they might be useful to address changes in thoughts and affect as associated with specific coaching session as well as their sequence in future research.

The differential observed phenomenological fingerprints related to thought content and emotional experience after different types of daily mental practices support the notion that the specific type and format of the practiced contemplative mental training matters [see also^4^]. Therefore, the choice of specific mental training can have a direct consequence on what our mind is occupied with, i.e., what we think and feel. Since thought content can be regarded as a precursor of subsequent mood changes^43^, fostering positive and pleasant thoughts may be a way of indirectly promoting psychological well-being and mental health long term^39–43^. However, based on our findings, 10 weeks of daily app-based practice did not yield longitudinal changes. Thus, future research might focus on thresholds of dosage and duration of contemplative training to achieve more long-term effects on thinking patterns.

While sociality and temporality of thought are not evaluated in their general desirability, specific patterns of thought content are characteristic of different mental disorders^44–46^. While it remains to be studied whether the ability to change these patterns using brief app-based mental trainings might even be helpful in generally redirecting thoughts away from destructive thinking patterns, such an approach might extend to several mental health conditions. In shifting the focus on positive and other-related thoughts, the Affect Dyad may be a particularly valuable tool in alleviating ruminative thinking, which is associated with strong negative, self-, and past-oriented thought content, and frequently occurs in major depressive disorder^44^. Alternatively, the ability to calm down an overly busy mind may generally help reduce symptom severity observed in other psychopathologies^67,68^. Previous studies have shown that repeated subjective experiences, such as feelings of anxiety^69^ or in contrast positive emotions^70^, have significant and far-reaching consequences for life, health, and longevity. As such, contemplative mental trainings may serve as an important instrument in helping to tone the subjective experiences away from those deemed harmful and noxious.

### Limitations

While the consistent and extensive measurement of participants’ subjective states through daily pre-post practice assessments in the context of participants’ everyday lives is a strength of this work, it retains the weaknesses which are common to self-report measures. Since participants were asked immediately before and after practice, retrospective biasing of responses could be reduced to a minimum. However, self-report measures are only viable if participants are able to self-reflect. Although we tried to correct for self-selection bias through the randomized controlled study design, the final sample included a higher percentage of women than men and had a higher monthly household income than the general population in Berlin, which may limit the generalizability of findings. It is noteworthy, however, that participants were originally recruited to participate in a COVID-19-related mental health study and not an intervention study and were thus relatively naïve to the present training methods and formed a rather heterogenous sample.

Occasionally, some participants of the socio-emotional training group in the first intervention phase encountered technical difficulties when trying to pair with their dyad partner, leading to disconnects and app-crashes. Although usually a stable connection could be established within a few tries, in rare cases participants were not able to resolve this issue for the day. This may have led to frustration and discouragement and thus may have diminished positive training effects. However, repeating the analyses using observations with only low counts of app crashes and aborts and high rates of compliance didn’t substantially change model estimates (see Supplement 4). While findings of change in thought patterns and affect valence and arousal were highly consistent for most outcome variables between the two socio-emotional interventions (SE and WSE) indicating the validity of the chosen approach, the statistical power to reliably detect changes over the 10 weeks intervention time may have been too low^71^. Thus, the approx. 10,000 data points on the Cube of Thought and Affect Grid for each outcome variable may still have been too few. Also, the use of single Likert-scale items to measure thought content and emotional well-being may have been insufficient to capture broad changes in these dimensions but more nuanced alterations may not have been successfully detected. Future research with more thorough instruments investigating the phenomenological space of these interventions might provide further insights. Furthermore, future studies with bigger sample sizes will be needed to test whether certain individuals benefit more from specific interventions than others, including sub-clinical and clinical populations.

## Conclusion

The current study investigated the differential effects of a rather novel daily app-based dyadic mental practice with socio-emotional focus as compared to app-delivered mindfulness-based practice on immediate changes and longitudinal changes over 10 weeks of training in thought patterns and emotional well-being. Indeed, we found evidence for the efficacy of both low-dose mental training approaches in an immediate improvement in affect valence after daily practice. Interestingly, findings revealed clear differential phenomenological fingerprints of both practices. While it could partly be replicated that solitary mindfulness practices are more effective in reducing thought content on several dimensions, the novel partner-based Affect Dyad was more powerful in increasing positive and other-related thoughts; the latter being in line with the social nature of this intersubjective socio-emotional practice. Whereas traditional in-person programs are limited to room sizes and local availability of teachers, app-delivered interventions are accessible, cheap, and can be learned within a comparably short time frame. The global scalability of these interventions offers immense potential in promoting mental health through changing patterns of thought and affect. Further, the unique practice-specific phenomenological fingerprints in thought patterns may help inform the development of personalized mental training programs tailored to the need of specific populations. These findings suggest promising avenues for scalable evidence-based brief mental intervention programs to improve psychological well-being and mitigate increasing mental health conditions that have been exacerbated during the COVID-19 pandemic.

### Supplementary Information

Supplementary Information.

## Data Availability

R code and results are available at an Open Science Framework repository (https://osf.io/vcasn/). The raw data sets analyzed during this study are not publicly available due to proprietary rights and data protection policies, but are available from the corresponding author on reasonable request.
