# Supplementary material for: A randomized trial on differential changes in thought and affect after mindfulness versus dyadic practice indicates phenomenological fingerprints of app-based interventions
Source: Sci Rep. 2023 Aug 24;13:13843. doi: 10.1038/s41598-023-40636-1 (PMC10449800; doi:10.1038/s41598-023-40636-1)
Supplement: Supplementary file 1 — Supplementary Information. [file 41598_2023_40636_MOESM1_ESM.pdf]

Supplemental Material

"A randomized trial on differential changes in thought and affect after mindfulness versus dyadic practice indicates phenomenological fingerprints of app-based interventions"

Supplement 1. A priori sample size calculation

An a-priori power analysis for repeated measures analyses of variance with between- and within-subject contrasts was conducted using G*Power [1]. This analysis was limited to the first intervention period (i.e., for changes from pretest to posttest 1), and was based on three groups, two measurements, significance level of α = .05, estimated power of .80, and repeated measures correlation of r = .39, which was constrained to the lowest retest reliability of outcome measures assessed in the project (i.e., Cortisol Awakening Response). Given a required sample size of n = 297 to detect small effect size of f = .10, we aimed to recruit 100 participants per intervention group.

Supplement 2: Example of the Affect Dayd in the CovSocial App

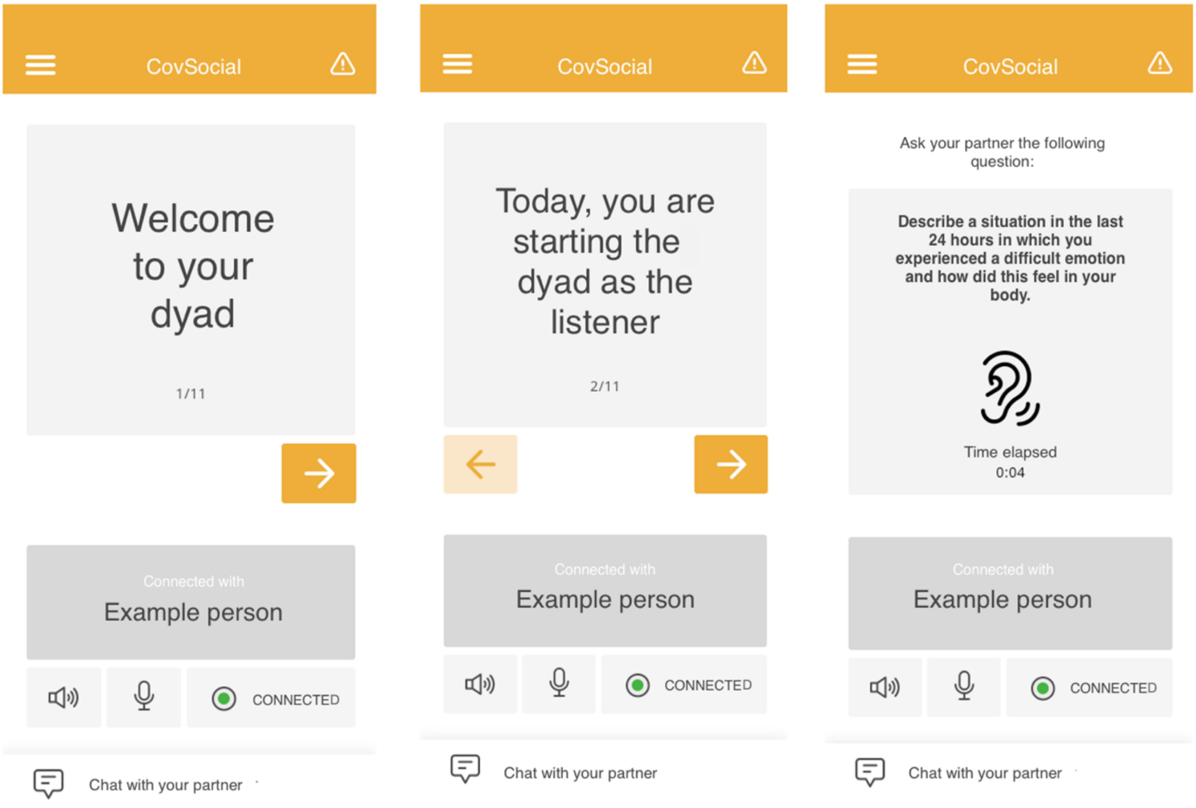

Supplement 3. Mental Training Intervention Protocol CovSocial Phase 2: Summary

In the following, key summary information is provided on the different weekly teaching sessions of the two intervention programs of the CovSocial project's phase 2. This standardized summary protocol document will be used as such in all publications of phase 2.

Teacher Training

Prior to the 10-week intervention, the four recruited mindfulness teachers underwent training on the specific format of the Affect Dyad from Prof. Dr. Tania Singer. Thus, in comparison to classic mindfulness intervention the four teachers were experts already as they were all certified in one or the other MBIs (MBCT, MBSR). The Affect Dyad as partner-based intersubjective mindfulness practice was not yet publicly available in commercial train-the-trainer programs. The four teachers practiced the Dyad daily among themselves and received weekly online coaching from Prof. Dr. Singer. Halfway through the coaching sessions, the teachers practiced the presentations themselves and received feedback from Prof. Dr. Singer. Another webinar was held to instruct the teachers in their roles and functions during the interventions. They also attended a three-day intensive in person workshop in Berlin to practice the presentation and receive in-depth information on scientific backgrounds for both intervention programs. All sessions were recorded and available to the teachers at any time.

Throughout the intervention phase, the trainers would regularly get feedback from Prof. Dr. Tania Singer or Birgit Severin who randomly attended some of the coaching sessions to ensure that the coaching material was being effectively and consistently communicated to both training groups. Additionally, supervision meetings were held at regular intervals between the teachers, Prof. Dr. Singer, and Birgit Severin to discuss any challenges or issues that arose within the groups or with the teaching materials, and to find solutions to them.

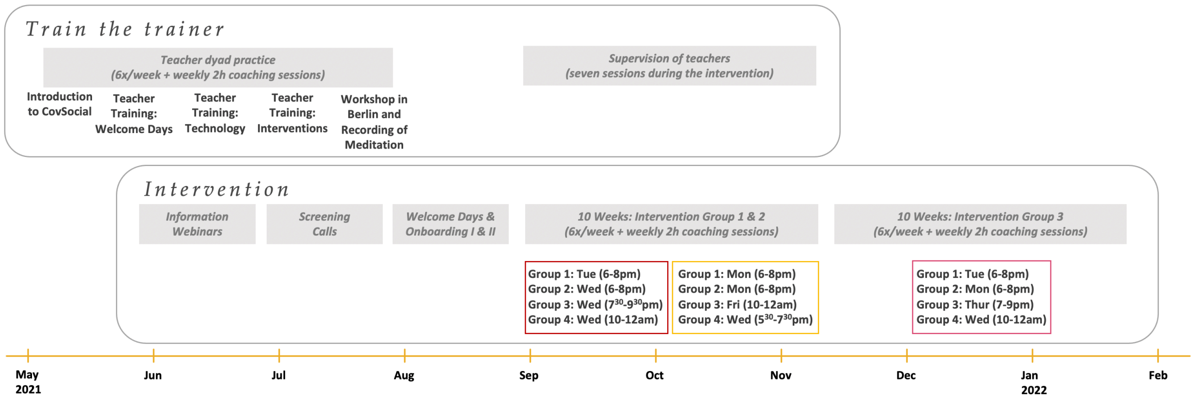

Welcome Days

The "Welcome Days" included several identical 90-minute online events via Webex, which were offered at different times of the day. The events consisted of two parts: a theoretical presentation and a practical introduction to demonstrate the trainings. Depending on whether participants were assigned to the socio-emotional or mindfulness-based training group, the presentations differed in content but followed the same overall structure. The theoretical part of the webinar introduced the team, addressed the first results of the CovSocial project phase 1, described the ReSource project and its scientific results, and built a bridge to the intervention phase 2. Detailed information on the schedule and study design was provided, and participants had the opportunity to ask questions before receiving further information in smaller, instructor-led subgroup sessions.

<u>Socio-emotional Training</u>

The second part of the webinar focused on the practical aspects of the socio-emotional training. Teachers who led the weekly coaching sessions briefly introduced themselves and explained the details of the Dyad exercise. The Dyad was also demonstrated with a CovSocial team member. At the end, participants had the opportunity to clarify any open questions.

<u>Mindfulness-based Training</u>

The second part of the webinar included a short introduction to mindfulness and meditation. Participants performed a breathing meditation under the guidance of teachers and had the opportunity to share and reflect on their experiences. They also received information on possible training effects and had time to address questions to the teachers.

Introductory Sessions („Kick-off“)

The intervention started with an information event ("kick-off") for all participants, providing an overview of mindfulness-based training techniques in a universal manner, without the need for separation by group. This event explained the contemplative context and scientific framework of classical mental training techniques like breathing meditation and body scan. Following this session, participants of the two interventions (socio-emotional training or mindfulness-based training) received individual coaching sessions tailored to each group.

Onboarding I & II

Both onboarding events (150 minutes each) took place within one week and were designed to provide participants with an understanding of the weekly coaching sessions and daily training

sessions that made up the group-specific online intervention. These webinars combined theory and practical parts to help participants gain a better understanding of the training objectives and clarify any questions they may have had. These sessions were also used to introduce participants already in the app and technology they would use for answering daily questions as part of the data assessment of the study. An in-person introduction to the app functions necessary for the daily practice was provided during the pre-test in the Social Neuroscience Lab. After Onboarding II, participants attended 10 weekly coaching sessions.

Socio-emotional training (Affect Dyad)

Onboarding I

During the first onboarding webinar (150 minutes), participants received an in-depth presentation on the details of the socio-emotional training. The webinar began with an introduction to motivational psychology, followed by a discussion of the hypotheses explored in the ReSource project, which introduced the Affect Dyad as one of several practices [2, 3]. The study design and relevant results of the ReSource project were then summarized, followed by an explanation of the "data collection" during the ten-week program of the CovSocial project. Participants had the opportunity to reflect on their motivations for participating in the training in breakout rooms, which were later discussed in the large group and the session concluded with five minutes of guided silence.

Onboarding II

During the second onboarding webinar (150 minutes), the participants were given a thorough explanation of the Dyad process. The webinar highlighted the importance of distinguishing between regular conversation and the specific rules of the contemplative Dyad practice, with particular emphasis on the Affect Dyad.

Coaching Sessions

Participants attended 2-hour long weekly coaching sessions for 10 weeks, on the same day and time each week in subgroups of 15-20 people per teacher. The coaching sessions followed a consistent structure, including sharing personal experiences, insights, and difficulties with the Dyad practice, as well as short presentations on various topics in order to deepen the practice. Every session started with a minute of silence and a check-in round. Afterwards, participants were split up into smaller groups to share in so-called "breakout rooms" their experiences with regards to each weeks' topic

and a sharing round with everyone. Then the teacher presented a scientific talk, followed by questions and answers as well as a second break-out room session. Each coaching ended with a minute of silence.

Coaching 1 – Framework of the Dyad

In the first weekly coaching session after participants had practiced their first week of daily Dyads at home, participants learned about the importance of establishing a routine and ritual for their daily socio-emotional practice, as well as creating a comfortable and quiet environment. They also discussed ways to address potential difficulties and obstacles, and the importance of self-commitment and consistency in their practice. Finally, they learned about the importance of stillness and the three minutes of silence at the beginning, middle and end of the Dyad practice, along with guidance on the dos and don'ts of the Dyad practice to ensure a psychologically safe space for all.

Coaching 2 – Learning About Body Language

During the second coaching session, participants were guided through the second part of the daily Dyad questions which involves asking how an unpleasant emotion or grateful experience felt in the body. They learned about the language of the body and how to develop a deeper awareness of bodily sensations. The session focused on understanding the relationship between cognitive concepts such as "I am happy" and the accompanying bodily sensations such as "my heart rate got calmer". This knowledge provided a vital foundation for the Dyad practice, which involved describing bodily sensations related to difficult emotions and gratitude on a daily basis to increase interoceptive body awareness.

Coaching 3 – Empathic Listening

During the third coaching session, the focus was on the active role of the listener in the Dyad, with a particular emphasis on empathy. Participants were introduced to the concept of empathy and the difference between empathy and compassion. They also learned how to practice empathic listening, including being fully present, releasing judgment, and listening with openness and acceptance. The benefits of empathic listening were discussed, including its ability to create space for concentration and benevolence, leading to deeper understanding and increased perceptions and perspectives.

Coaching 4 – Gratitude

In the fourth coaching session, the topic of gratitude and its impact on our well-being was discussed, with a focus on the practical application of the Dyad practice in daily life. The following topics were

covered: defining gratitude, recognizing obstacles and challenges in cultivating gratitude, exploring the positive effects of gratitude, understanding how to recognize gratitude and differentiate it from other pleasant emotion, and discussing different examples for which we can be grateful.

Coaching 5 – Difficult Emotions

During the fifth coaching session, the Dyad practice focused on exploring negative emotions. The session began with a discussion of emotions in general, including their functions and how they can lead to different adaptive or non-adaptive reactions. The importance of accepting negative emotions by becoming aware of them and neither dumping them on others nor suppressing or altering them was emphasized. The session offered coping strategies such as observing and embracing the emotions to reduce suffering.

Coaching 6 – The Dyad – a Special Form of Social Interaction

In the sixth coaching session, the focus was on the partner-based and social format of contemplative Dyads. The session covered the specificities of this interaction, which does not allow for a real dialogue. Participants were reminded of the specific behavioral rules and goals of this daily Dyad practice, such as improving attention, awareness of bodily sensations, empathetic and non-judgmental listening, and accepting difficult emotions and stress while building resilience through gratitude, shared humanity, tolerance, and calmness. Additionally, triggers for each role were discussed, and strategies for handling partner absence or other social triggers were shared.

Coaching 7 – Change, a Beginner's Mind and Unintentionality

The seventh coaching session delved into the changes that occur over time with daily practice. Topics covered included the plateau effect, the importance of maintaining a beginner's mind, practicing with non-intentionality, and deepening one's experience by changing the focus to different aspects of bodily sensations and triggers. The session emphasized the need to approach practice with an open and curious attitude, free of judgment and preconceptions, in order to continue to learn and grow.

Coaching 8 – Recurring Patterns and the Dyad Practice

In the eighth coaching session, participants learned about psychological patterns and schemas, and focused on identifying recurring patterns in their thoughts, feelings, and actions, both within and outside of the socio-emotional Dyad training. They explored the definition of patterns and how they affect their daily Dyad practice, such as how they tend to deal with negative emotions or express

gratitude. Additionally, participants reflected on their patterns when dealing with silence and throughout practicing affect Dyads in general.

Coaching 9 – How does the Dyad Transfer into my Everyday Life?

In the ninth coaching session, participants were prompted to reflect on how the socio-emotional training had impacted their daily lives and extended beyond the Dyad practice. They were encouraged to contemplate the changes they noticed in various aspects such as self-awareness, bodily sensations, coping with difficult emotions, and their ability to listen, empathize, change perspectives, and be open to others. Additionally, participants were encouraged to reflect on any shifts in their goals and priorities in life that might have emerged as a result of their practice.

Coaching 10 – The Last Time

In the tenth and final coaching session, participants engaged in a Dyad exercise and a closing ritual, in addition to a presentation. The presentation emphasized the potential positive impact of further training, and participants were encouraged to continue if they wished. To reflect on the training, participants were asked to answer questions regarding the biggest challenges and the value of the Dyad, and then shared their conclusions via Webex chat.

Mindfulness-based Training

Onboarding I

The first onboarding webinar (150 minutes) provided an in-depth introduction to the mindfulness-based training, starting with an overview of the "ReSource Project" and its findings on attention, body awareness, interoceptive accuracy, and subjective well-being. The CovSocial project's intervention phase was also introduced, along with information on data collection during the ten-week program. To clarify participants' motives for joining, they were given the opportunity to discuss their motivations in small groups. The different meditation formats in the intervention, such as breathing and open awareness meditation, were briefly explained and practiced. The session concluded with a discussion on how to handle difficulties, along with guidance of dos and don'ts.

Onboarding II

During the second onboarding webinar (150 minutes), the focus was on deepening the breathing meditation practice to prepare participants for the upcoming four-week daily practice. The

importance of body awareness was emphasized through a body scan exercise. The session ended with a guided reflection on how to integrate the mindfulness training into everyday life and an introduction to the CovSocial app.

Coaching Sessions

Participants attended 2-hour long weekly coaching sessions for 10 weeks, on the same day each week. The coaching sessions followed a consistent structure with some slight variations in timing and included sharing personal experiences, and short presentations on various topics. The structure of the coaching was exactly the same as in the dyad coachings in order to making them comparable across intervention.

Coaching 1 – Breathing Meditation

During the first coaching session, common difficulties associated with the first classical mindfulness practice of breathing meditation were discussed, including challenges in maintaining focus on the breath, lack of motivation, and unrealistic expectations. Strategies for overcoming these obstacles were presented, and the importance of having a clear structure or routine for the mindfulness practice was emphasized.

Coaching 2 – Body Awareness

The second coaching session focused on how bodily sensations can be used to anchor oneself in the present moment during mindfulness practice. Participants were taught about the interconnection between the body and mind and were guided on how to deepen their body awareness by recognizing the differences between sensations, feelings, and thoughts.

Coaching 3 – Dealing with Difficulties in Mindfulness Training

In the third coaching session, participants were provided with strategies to overcome common challenges that may arise during mindfulness practice, such as restlessness, fatigue, aversion, cravings, doubts, and other obstacles. The session introduced the *RAIN* concept as a helpful tool to manage these difficulties, which involves *recognizing* the issue, *accepting* it, *investigating* it, and practicing *non-identification*.

Coaching 4 – Training Sensory Awareness – Hearing

In the fourth coaching session, participants were introduced to a new type of meditation: the meditation on sounds. The session covered various topics related to sensory stimuli and mindfulness

through sense perception, including the interpretation and evaluation of auditive stimuli. The session also included guidance on how to train mindfulness of sounds (meditation on sounds) by perceiving sounds without interpretation or conceptualization. This was followed by a group discussion on participants' personal experiences.

Coaching 5 – Training Sensory Awareness – Seeing

The fifth coaching session expanded the topic of sensory perception to include vision and explored whether it is possible to observe visual stimuli without the interference of interpretations and judgments. The session emphasized the intention to stabilize the mind and increase awareness and discussed general goals such as becoming more present and focused, broadening perspective, perceiving non-judgmentally, and reducing one's own reactivity. To illustrate these concepts, a vision meditation was introduced, and a 12-minute practice session was conducted, followed by a group discussion.

Coaching 6 – Mindfulness in all Senses

The sixth coaching session focused on the five senses and included the "classic" raisin exercise, which aims to explore all senses - seeing, feeling, hearing, smelling, and tasting. Participants practiced eating a raisin with guidance from the teacher, then independently exploring it, and finally eating it normally. The session ended with a discussion of the experiences and how to apply mindfulness to everyday life or mindful eating.

Coaching 7 – Open Awareness

The seventh coaching introduced a new kind of meditation called "open awareness," where the meditator is open to all sensations without focusing on a particular object of attention (e.g., breath, sounds) or holding onto them. The focus is on open awareness itself, with the breath serving as an anchor in the present moment. Participants explored their first experiences with this type of meditation in group exercises guided by the teacher.

Coaching 8 – Stress and Mindfulness

The eighth coaching session focused on stress, including its definition, psychological reactions, causes, and consequences. The relationship between stress and breath was elaborated to highlight the benefits of mindfulness training in everyday life.

Coaching 9 – How Can We Transfer Mindfulness Training into Everyday Life?

The ninth coaching emphasized on the integration of mindfulness practice into daily life. Participants were encouraged to reflect on their progress, experiences, and changes, such as how their practice has developed, the impact of the training on their daily lives, their relationships, and whether there are noticeable changes in their reactions and interpretations, which may influence their goals in life.

Coaching 10 – The Last Time

In the tenth and final coaching session of the mindfulness training, participants engaged in exchanging experiences and a closing ritual, in addition to a presentation. Participants were invited to continue their training and asked to reflect on how the mindfulness training changed over the 10 weeks and how it affected their everyday lives. They then shared their conclusions via chat, which were read out by the teachers, and ended the session with a closing ritual involving a personal object symbolic of the shared journey.

End of Intervention

After the ten weeks of intervention, participants were invited to voluntarily keep using the app for another 10 weeks without coaching sessions. In order to complete the post-test, participants underwent the same assessments as for pre-test for 2 weeks after the last coaching sessions. Participants had the option to take part in the TSST, which was conducted during an additional appointment at the lab.

Supplement 4. Bayesian hierarchical models

Following recommendations of Liddell and Kruschke [4] regarding the analysis of (single) Likert-scale items, we adopted an ordinal Bayesian mixed modelling approach [5, 6] for each dependent variable using the R-package *brms* [7].

Model specifications

Model 1: Linear model

```
model1 = brm(DV ~ intervention * occasion * tp, family = cumulative("probit"))
```

Model 2: Random intercept model

```
model2 = brm(DV ~ intervention * occasion * time + (1 | id),
             family = cumulative("probit"))
```

Model 3: Random intercept and slope model with random slope for *occasion*

```
model3 = brm(DV ~ intervention * occasion * time + (1 + occasion | id),
             family = cumulative("probit"))
```

Model 4: Random intercept and slope model with random slopes for *occasion* x *time*

```
model4 = brm(DV ~ intervention * occasion * time + (1 + occasion + time | id),
             family = cumulative("probit"))
```

Model 5: Models for step2 (final parameter estimation) additionally included covariates (age, sex, weekend, motivation)

```
model4 = brm(DV ~ COVARIATES + intervention * occasion * time +
             (1 + occasion + time | id),
             family = cumulative("probit"))
```

Prior distributions

Weakly informative priors for the linear models

```
prior1 <- c(
  prior(normal(0, 1), class = "Intercept"),
  prior(normal(0, 1), class = "b")
)
```

Weakly informative priors for the random intercept models

```
prior2 <- c(
  prior(normal(0, 1), class = "Intercept"),
  prior(normal(0, 1), class = "b"),
  prior(student_t(4, 0, 1), class = "sd")
)
```

Weakly informative priors for random intercept and slope models

```
prior3 <- c(
  prior(normal(0, 1), class = "Intercept"),
  prior(normal(0, 1), class = "b"),
  prior(student_t(4, 0, 1), class = "sd"),
  prior(lkj(2), class = cor)
)
```

Super weakly informative priors for random intercept and slope models

```
prior3_2 <- c(
  prior(normal(0, 10), class = "Intercept"),
  prior(normal(0, 10), class = "b"),
  prior(student_t(4, 0, 10), class = "sd"),
  prior(lkj(2), class = cor)
)
```

Supplement 4. Model selection and parameter estimates

Table S1. Spearman-correlation matrix of all assessed outcome variables

	cot1	cot2	cot3	cot4	cot5	cot6	val	aro
cot1	**0.39**	0.03	0.21	0.24	0.18	0.01	0.05	-0.15
cot2	-0.08	**0.36**	0.19	0.20	-0.08	0.30	-0.10	-0.05
cot3	0.02	0.15	**0.27**	-0.05	0.13	0.04	0.07	0.00
cot4	0.14	0.34	0.12	**0.30**	0.05	0.15	-0.02	-0.09
cot5	0.08	-0.02	0.27	0.10	**0.49**	-0.61	0.62	0.07
cot6	0.00	0.37	0.03	0.22	-0.45	**0.51**	-0.56	-0.11
val	0.03	-0.10	0.14	-0.01	0.47	-0.40	**0.66**	0.23
aro	-0.07	-0.06	0.09	-0.14	0.06	-0.09	0.24	**0.58**

Note. The immediate pre-intervention correlations are presented above the diagonal; the immediate post-intervention correlations are presented below the diagonal; the correlations of each variable between pre-and post-intervention are presented in bold on the diagonal. cot1 = Thoughts about future, cot2 = Thoughts about past, cot3 = Thoughts about self, cot4 = Thoughts about others, cot5 = Positive thoughts, cot6 = Negative Thoughts, val = Affect valence, aro = Arousal.

Table S2. Bayesian model evaluation using leave-one-out cross validation

cot1 – Future	elpd_loo	elpd_diff	se_diff
model4	-12391.7	0.0	0.0
model3	-12549.2	-157.6	19.1
model2	-12814.5	-422.8	31.6
model1	-14502.1	-2110.5	63.8

cot2 – Past	elpd_loo	elpd_diff	se_diff
model4	-11574.7	0.0	0.0
model3	-11793.9	-219.2	23.5
model2	-12199.7	-625.0	38.5
model1	-13529.0	-1954.3	64.1

cot3 – Self	elpd_loo	elpd_diff	se_diff
model4	-10888.1	0.0	0.0
model3	-11021.4	-133.3	17.2
model2	-11459.6	-571.6	37.4
model1	-12720.5	-1832.4	62.0

cot4 – Other	elpd_loo	elpd_diff	se_diff
model4	-11800.4	0.0	0.0
model3	-11924.0	-123.6	16.9
model2	-12183.8	-383.4	29.2
model1	-13364.6	-1564.2	55.6

cot5 – Positive	elpd_loo	elpd_diff	se_diff
model4	-10311.1	0.0	0.0
model3	-10396.5	-85.4	13.5
model2	-10444.2	-133.1	17.1
model1	-12071.7	-1760.6	58.7

cot6 – Negative	elpd_loo	elpd_diff	se_diff
model4	-10865.2	0.0	0.0
model3	-10977.5	-112.2	15.8
model2	-11014.7	-149.4	18.5
model1	-12470.2	-1605.0	54.7

ag – Valence	elpd_loo	elpd_diff	se_diff
model4	-16071.9	0.0	0.0
model3	-16216.8	-144.9	18.3
model2	-16306.3	-234.4	23.3
model1	-18283.6	-2211.7	66.6

ag – Arousal	elpd_loo	elpd_diff	se_diff
model4	-17210.7	0.0	0.0
model3	-17297.3	-86.6	14.4
model2	-17381.5	-170.8	20.1
model1	-18762.9	-1552.2	59.6

Note. elpd_loo is the Bayesian LOO estimate of the expected log pointwise predictive density. elpd_diff is the difference in elpd_loo for two models; The difference is computed relative to the model with the highest elpd_loo. se_diff is the standard error component-wise differences of elpd_loo between two models (for further information, see [8]). cot = Cube of Thought, ag = Affect Grid.

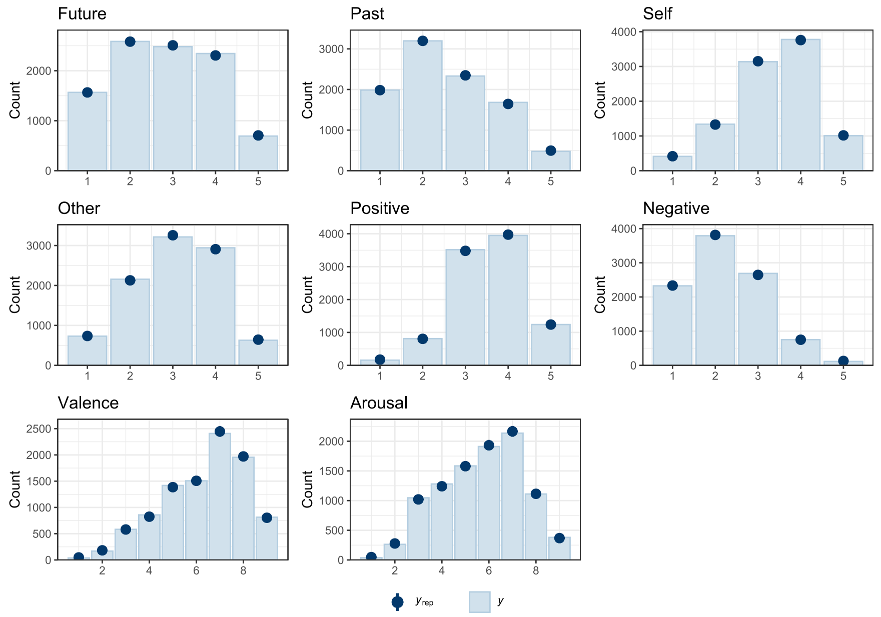

Figure S1. Posterior predictive checks**.** Comparison between data generated by the posterior predictive distribution and observed data.

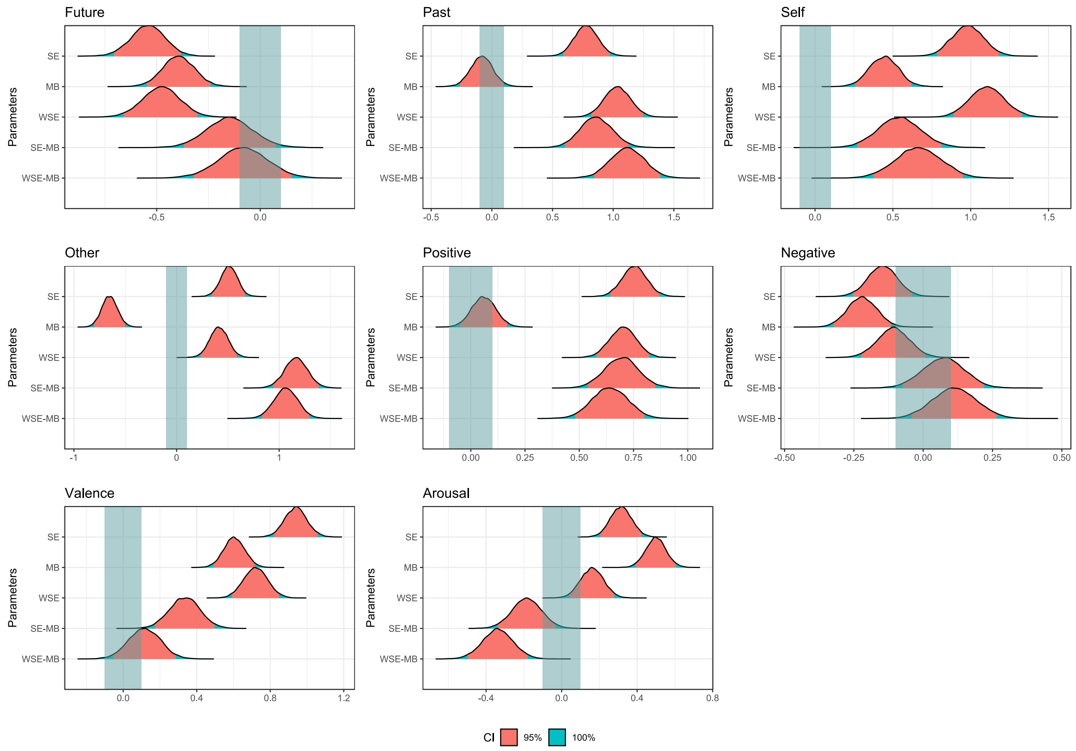

Figure S2. Posterior distributions of absolute and differential immediate pre-post practice state changes. Posterior distributions of absolute immediate pre-post practice state changes for the socio-emotional training (SE/WSE) and mindfulness-based training (MB) and differential effects between the socio-emotional trainings and mindfulness-based training (SE-MB, WSE-MB). The marked area in blue depicts the region of practical equivalence (ROPE).

Table S3. Results of the Multilevel Analyses of each outcome variable using weakly, super weakly informative priors and only for observations with compliance and low app crashes and aborts.

CoT - Future	Weakly informative priors				Super weakly informative priors				High compliance, low aborts			
	b	95%-CI	$SD_{Subjects}$	95%-CI	*b*	95%-CI	$SD_{Subjects}$	95%-CI	*b*	95%-CI	$SD_{Subjects}$	95%-CI
Intercept			0.74	[0.66, 0.82]			0.74	[0.66, 0.83]			0.74	[0.65, 0.84]
Age	-0.09	[-0.19, 0.01]			-0.09	[-0.19, 0.01]			-0.10	[-0.21, 0.02]		
Sex	-0.13	[-0.36, 0.10]			-0.14	[-0.37, 0.10]			-0.05	[-0.30, 0.21]		
Motivation	0.10	[0.06, 0.13]			0.10	[0.06, 0.13]			0.09	[0.05, 0.13]		
Weekend	-0.04	[-0.09, 0.00]			-0.04	[-0.09, 0.00]			-0.05	[-0.10, 0.00]		
SE	0.01	[-0.23, 0.26]			0.00	[-0.25, 0.24]			-0.02	[-0.30, 0.26]		
WSE	-0.48	[-0.73, -0.24]			-0.49	[-0.75, -0.23]			-0.55	[-0.85, -0.25]		
Occasion	-0.39	[-0.55, -0.24]	0.62	[0.55, 0.71]	-0.40	[-0.55, -0.24]	0.62	[0.55, 0.71]	-0.41	[-0.59, -0.22]	0.64	[0.55, 0.73]
Time	-0.07	[-0.14, 0.01]	0.25	[0.21, 0.28]	-0.07	[-0.14, 0.01]	0.25	[0.21, 0.28]	-0.07	[-0.16, 0.02]	0.25	[0.21, 0.29]
SE: Occasion	-0.15	[-0.37, 0.07]			-0.14	[-0.36, 0.08]			-0.23	[-0.49, 0.02]		
WSE: Occasion	-0.08	[-0.32, 0.15]			-0.08	[-0.32, 0.16]			-0.05	[-0.31, 0.22]		
SE:Time	-0.01	[-0.12, 0.10]			-0.01	[-0.12, 0.10]			-0.05	[-0.18, 0.07]		
WSE:Time	0.07	[-0.04, 0.19]			0.07	[-0.04, 0.19]			0.07	[-0.06, 0.20]		
Occasion:Time	-0.01	[-0.08, 0.06]			-0.01	[-0.08, 0.06]			-0.02	[-0.11, 0.06]		
SE:Occasion:Time	0.17	[0.07, 0.28]			0.17	[0.07, 0.28]			0.21	[0.09, 0.32]		
WSE:Occasion:Time	0.06	[-0.05, 0.17]			0.06	[-0.05, 0.17]			0.08	[-0.04, 0.20]		

CoT - Past	Weakly informative priors				Super weakly informative priors				High compliance, low aborts			
	b	95%-CI	$SD_{Subjects}$	95%-CI	*b*	95%-CI	$SD_{Subjects}$	95%-CI	*b*	95%-CI	$SD_{Subjects}$	95%-CI
Intercept			0.65	[0.58, 0.73]			0.65	[0.58, 0.73]			0.66	[0.58, 0.75]
Age	-0.06	[-0.16, 0.04]			-0.06	[-0.16, 0.03]			-0.02	[-0.13, 0.09]		
Sex	-0.21	[-0.42, 0.00]			-0.22	[-0.43, -0.01]			-0.21	[-0.46, 0.04]		
Motivation	0.06	[0.02, 0.09]			0.06	[0.02, 0.09]			0.07	[0.03, 0.11]		
Weekend	-0.02	[-0.06, 0.03]			-0.02	[-0.06, 0.03]			-0.03	[-0.08, 0.03]		
SE	0.64	[0.42, 0.86]			0.64	[0.43, 0.87]			0.62	[0.36, 0.88]		
WSE	0.34	[0.11, 0.57]			0.34	[0.11, 0.58]			0.27	[0.01, 0.54]		
Occasion	-0.08	[-0.26, 0.10]	0.76	[0.68, 0.86]	-0.10	[-0.29, 0.08]	0.76	[0.68, 0.86]	-0.08	[-0.31, 0.15]	0.82	[0.72, 0.93]
Time	-0.02	[-0.10, 0.06]	0.28	[0.25, 0.33]	-0.02	[-0.11, 0.06]	0.28	[0.25, 0.32]	-0.02	[-0.12, 0.07]	0.28	[0.24, 0.33]
SE: Occasion	0.86	[0.60, 1.13]			0.88	[0.63, 1.15]			0.82	[0.50, 1.14]		
WSE: Occasion	1.12	[0.84, 1.39]			1.15	[0.86, 1.42]			1.10	[0.78, 1.43]		
SE:Time	-0.04	[-0.15, 0.08]			-0.03	[-0.15, 0.08]			-0.05	[-0.18, 0.09]		
WSE:Time	-0.03	[-0.15, 0.09]			-0.03	[-0.15, 0.09]			-0.01	[-0.15, 0.13]		
Occasion:Time	-0.07	[-0.14, 0.01]			-0.07	[-0.14, 0.01]			-0.10	[-0.18, -0.01]		
SE:Occasion:Time	0.09	[-0.01, 0.20]			0.09	[-0.01, 0.20]			0.14	[0.02, 0.26]		
WSE:Occasion:Time	-0.01	[-0.13, 0.10]			-0.01	[-0.13, 0.10]			0.01	[-0.11, 0.13]		

CoT - Self	Weakly informative priors				Super weakly informative priors				High compliance, low aborts			
	b	95%-CI	$SD_{Subjects}$	95%-CI	*b*	95%-CI	$SD_{Subjects}$	95%-CI	*b*	95%-CI	$SD_{Subjects}$	95%-CI
Intercept			0.72	[0.65, 0.81]			0.73	[0.65, 0.81]			0.73	[0.65, 0.83]
Age	0.03	[-0.06, 0.12]			0.03	[-0.07, 0.12]			0.08	[-0.03, 0.18]		
Sex	-0.20	[-0.41, 0.01]			-0.20	[-0.41, 0.01]			-0.13	[-0.38, 0.11]		
Motivation	0.13	[0.10, 0.16]			0.13	[0.10, 0.16]			0.12	[0.08, 0.16]		
Weekend	-0.02	[-0.07, 0.03]			-0.02	[-0.06, 0.03]			-0.01	[-0.06, 0.05]		
SE	0.27	[0.03, 0.51]			0.27	[0.03, 0.51]			0.36	[0.08, 0.64]		
WSE	0.11	[-0.15, 0.36]			0.11	[-0.15, 0.37]			0.17	[-0.12, 0.46]		
Occasion	0.44	[0.26, 0.62]	0.79	[0.70, 0.88]	0.43	[0.25, 0.62]	0.79	[0.70, 0.88]	0.35	[0.13, 0.56]	0.76	[0.67, 0.87]
Time	0.07	[0.00, 0.15]	0.23	[0.19, 0.26]	0.07	[0.00, 0.15]	0.23	[0.19, 0.26]	0.08	[0.00, 0.17]	0.23	[0.20, 0.27]
SE: Occasion	0.54	[0.27, 0.81]			0.56	[0.28, 0.82]			0.58	[0.28, 0.88]		
WSE: Occasion	0.66	[0.38, 0.95]			0.68	[0.39, 0.97]			0.76	[0.45, 1.07]		
SE:Time	-0.09	[-0.19, 0.02]			-0.09	[-0.19, 0.02]			-0.10	[-0.22, 0.02]		
WSE:Time	-0.02	[-0.13, 0.09]			-0.02	[-0.13, 0.09]			-0.02	[-0.15, 0.10]		
Occasion:Time	-0.21	[-0.29, -0.14]			-0.21	[-0.29, -0.14]			-0.23	[-0.31, -0.15]		
SE:Occasion:Time	0.20	[0.09, 0.30]			0.20	[0.09, 0.31]			0.21	[0.09, 0.32]		
WSE:Occasion:Time	0.10	[-0.01, 0.21]			0.10	[-0.01, 0.21]			0.09	[-0.03, 0.21]		

CoT - Other	Weakly informative priors				Super weakly informative priors				High compliance, low aborts			
	b	95%-CI	$SD_{Subjects}$	95%-CI	*b*	95%-CI	$SD_{Subjects}$	95%-CI	*b*	95%-CI	$SD_{Subjects}$	95%-CI
Intercept			0.62	[0.55, 0.70]			0.62	[0.56, 0.70]			0.62	[0.55, 0.70]
Age	-0.01	[-0.10, 0.07]			-0.02	[-0.11, 0.07]			-0.03	[-0.13, 0.07]		
Sex	-0.27	[-0.46, -0.08]			-0.27	[-0.47, -0.08]			-0.15	[-0.36, 0.08]		
Motivation	0.06	[0.02, 0.09]			0.06	[0.02, 0.09]			0.06	[0.02, 0.10]		
Weekend	0.03	[-0.02, 0.07]			0.03	[-0.02, 0.07]			0.05	[0.00, 0.11]		
SE	0.24	[0.03, 0.45]			0.23	[0.02, 0.45]			0.26	[0.02, 0.50]		
WSE	0.00	[-0.22, 0.21]			-0.01	[-0.24, 0.21]			-0.04	[-0.28, 0.21]		
Occasion	-0.65	[-0.80, -0.50]	0.61	[0.53, 0.69]	-0.67	[-0.82, -0.52]	0.61	[0.54, 0.69]	-0.63	[-0.82, -0.44]	0.64	[0.55, 0.73]
Time	-0.13	[-0.20, -0.06]	0.22	[0.19, 0.26]	-0.13	[-0.20, -0.06]	0.22	[0.19, 0.26]	-0.16	[-0.24, -0.08]	0.22	[0.18, 0.26]
SE: Occasion	1.16	[0.94, 1.39]			1.19	[0.97, 1.41]			1.17	[0.91, 1.44]		
WSE: Occasion	1.07	[0.83, 1.30]			1.10	[0.86, 1.33]			1.10	[0.83, 1.38]		
SE:Time	0.06	[-0.05, 0.16]			0.06	[-0.04, 0.17]			0.07	[-0.04, 0.19]		
WSE:Time	0.09	[-0.02, 0.20]			0.09	[-0.01, 0.20]			0.16	[0.05, 0.28]		
Occasion:Time	0.04	[-0.03, 0.11]			0.04	[-0.03, 0.11]			0.05	[-0.03, 0.14]		
SE:Occasion:Time	0.05	[-0.05, 0.15]			0.05	[-0.06, 0.15]			0.05	[-0.07, 0.17]		
WSE:Occasion:Time	-0.05	[-0.16, 0.05]			-0.06	[-0.16, 0.05]			-0.11	[-0.22, 0.01]		

CoT - Positive	Weakly informative priors				Super weakly informative priors				High compliance, low aborts			
	b	95%-CI	$SD_{Subjects}$	95%-CI	*b*	95%-CI	$SD_{Subjects}$	95%-CI	*b*	95%-CI	$SD_{Subjects}$	95%-CI
Intercept			0.76	[0.68, 0.86]			0.77	[0.69, 0.86]			0.69	[0.60, 0.78]
Age	0.07	[-0.04, 0.17]			0.07	[-0.04, 0.17]			0.06	[-0.05, 0.16]		
Sex	-0.03	[-0.27, 0.20]			-0.03	[-0.28, 0.20]			-0.01	[-0.25, 0.23]		
Motivation	0.33	[0.30, 0.37]			0.33	[0.30, 0.37]			0.35	[0.31, 0.39]		
Weekend	0.09	[0.04, 0.14]			0.09	[0.04, 0.14]			0.11	[0.05, 0.16]		
SE	-0.22	[-0.47, 0.03]			-0.23	[-0.48, 0.03]			-0.21	[-0.48, 0.06]		
WSE	-0.39	[-0.66, -0.11]			-0.40	[-0.67, -0.13]			-0.31	[-0.59, -0.03]		
Occasion	0.06	[-0.05, 0.17]	0.35	[0.28, 0.42]	0.05	[-0.05, 0.16]	0.35	[0.28, 0.42]	0.02	[-0.11, 0.15]	0.37	[0.30, 0.45]
Time	-0.02	[-0.09, 0.05]	0.19	[0.16, 0.23]	-0.02	[-0.09, 0.05]	0.19	[0.16, 0.23]	0.01	[-0.07, 0.08]	0.18	[0.14, 0.22]
SE: Occasion	0.69	[0.54, 0.85]			0.70	[0.55, 0.86]			0.78	[0.59, 0.96]		
WSE: Occasion	0.64	[0.48, 0.80]			0.65	[0.49, 0.82]			0.67	[0.48, 0.86]		
SE:Time	0.01	[-0.09, 0.10]			0.01	[-0.09, 0.10]			-0.02	[-0.13, 0.09]		
WSE:Time	0.08	[-0.02, 0.18]			0.08	[-0.02, 0.18]			0.07	[-0.05, 0.18]		
Occasion:Time	-0.01	[-0.09, 0.06]			-0.02	[-0.09, 0.06]			-0.05	[-0.13, 0.04]		
SE:Occasion:Time	-0.01	[-0.12, 0.09]			-0.01	[-0.12, 0.09]			0.01	[-0.11, 0.13]		
WSE:Occasion:Time	-0.08	[-0.19, 0.04]			-0.08	[-0.18, 0.03]			-0.06	[-0.19, 0.07]		

CoT - Negative	Weakly informative priors				Super weakly informative priors				High compliance, low aborts			
	b	95%-CI	$SD_{Subjects}$	95%-CI	*b*	95%-CI	$SD_{Subjects}$	95%-CI	*b*	95%-CI	$SD_{Subjects}$	95%-CI
Intercept			0.73	[0.65, 0.81]			0.73	[0.65, 0.82]			0.68	[0.60, 0.78]
Age	-0.15	[-0.25, -0.05]			-0.15	[-0.25, -0.05]			-0.15	[-0.26, -0.04]		
Sex	-0.19	[-0.43, 0.04]			-0.19	[-0.42, 0.04]			-0.16	[-0.40, 0.09]		
Motivation	-0.17	[-0.20, -0.14]			-0.17	[-0.20, -0.14]			-0.16	[-0.20, -0.13]		
Weekend	-0.10	[-0.15, -0.05]			-0.10	[-0.15, -0.05]			-0.13	[-0.18, -0.07]		
SE	0.59	[0.35, 0.84]			0.60	[0.36, 0.85]			0.63	[0.37, 0.90]		
WSE	0.57	[0.32, 0.83]			0.58	[0.33, 0.84]			0.51	[0.22, 0.79]		
Occasion	-0.22	[-0.32, -0.12]	0.32	[0.25, 0.38]	-0.22	[-0.33, -0.11]	0.32	[0.25, 0.38]	-0.24	[-0.36, -0.11]	0.31	[0.24, 0.39]
Time	-0.13	[-0.20, -0.05]	0.23	[0.19, 0.27]	-0.13	[-0.20, -0.05]	0.23	[0.20, 0.27]	-0.15	[-0.23, -0.06]	0.21	[0.17, 0.25]
SE: Occasion	0.07	[-0.07, 0.22]			0.08	[-0.07, 0.23]			0.04	[-0.13, 0.20]		
WSE: Occasion	0.11	[-0.04, 0.27]			0.11	[-0.04, 0.27]			0.15	[-0.03, 0.32]		
SE:Time	0.14	[0.03, 0.24]			0.14	[0.03, 0.24]			0.15	[0.04, 0.27]		
WSE:Time	0.04	[-0.07, 0.16]			0.05	[-0.07, 0.16]			0.08	[-0.04, 0.20]		
Occasion:Time	0.01	[-0.06, 0.09]			0.01	[-0.06, 0.09]			0.02	[-0.07, 0.11]		
SE:Occasion:Time	-0.05	[-0.16, 0.06]			-0.05	[-0.16, 0.05]			-0.06	[-0.19, 0.06]		
WSE:Occasion:Time	0.03	[-0.08, 0.15]			0.03	[-0.08, 0.14]			0.02	[-0.11, 0.14]		

AG - Valence	Weakly informative priors				Super weakly informative priors				High compliance, low aborts			
	b	95%-CI	$SD_{Subjects}$	95%-CI	*b*	95%-CI	$SD_{Subjects}$	95%-CI	*b*	95%-CI	$SD_{Subjects}$	95%-CI
Intercept			0.82	[0.74, 0.91]			0.82	[0.74, 0.92]			0.78	[0.69, 0.88]
Age	0.06	[-0.05, 0.17]			0.06	[-0.05, 0.17]			0.05	[-0.07, 0.17]		
Sex	-0.14	[-0.40, 0.11]			-0.15	[-0.40, 0.10]			-0.11	[-0.38, 0.16]		
Motivation	0.36	[0.33, 0.39]			0.36	[0.33, 0.39]			0.36	[0.33, 0.40]		
Weekend	0.22	[0.17, 0.26]			0.22	[0.17, 0.26]			0.24	[0.19, 0.29]		
SE	-0.29	[-0.56, -0.02]			-0.29	[-0.56, -0.02]			-0.28	[-0.59, 0.01]		
WSE	-0.35	[-0.62, -0.06]			-0.36	[-0.65, -0.07]			-0.28	[-0.58, 0.03]		
Occasion	0.60	[0.49, 0.72]	0.41	[0.35, 0.47]	0.61	[0.50, 0.72]	0.41	[0.35, 0.47]	0.65	[0.51, 0.80]	0.44	[0.37, 0.51]
Time	-0.01	[-0.08, 0.06]	0.23	[0.19, 0.26]	-0.01	[-0.08, 0.06]	0.23	[0.20, 0.27]	0.00	[-0.08, 0.08]	0.21	[0.17, 0.25]
SE: Occasion	0.33	[0.17, 0.50]			0.34	[0.17, 0.50]			0.33	[0.13, 0.53]		
WSE: Occasion	0.12	[-0.05, 0.29]			0.12	[-0.05, 0.29]			0.06	[-0.14, 0.26]		
SE:Time	0.05	[-0.05, 0.15]			0.05	[-0.05, 0.15]			0.06	[-0.05, 0.17]		
WSE:Time	0.05	[-0.06, 0.15]			0.05	[-0.06, 0.16]			0.04	[-0.07, 0.15]		
Occasion:Time	-0.03	[-0.10, 0.04]			-0.03	[-0.10, 0.04]			-0.05	[-0.13, 0.03]		
SE:Occasion:Time	-0.02	[-0.13, 0.08]			-0.03	[-0.13, 0.08]			-0.01	[-0.12, 0.11]		
WSE:Occasion:Time	-0.05	[-0.15, 0.05]			-0.05	[-0.15, 0.05]			-0.03	[-0.15, 0.08]		

AG - Arousal	Weakly informative priors				Super weakly informative priors				High compliance, low aborts			
	b	95%-CI	$SD_{Subjects}$	95%-CI	*b*	95%-CI	$SD_{Subjects}$	95%-CI	*b*	95%-CI	$SD_{Subjects}$	95%-CI
Intercept			0.64	[0.57, 0.72]			0.64	[0.57, 0.72]			0.64	[0.57, 0.72]
Age	-0.02	[-0.12, 0.07]			-0.03	[-0.12, 0.07]			-0.03	[-0.12, 0.07]		
Sex	-0.03	[-0.23, 0.17]			-0.03	[-0.23, 0.18]			-0.03	[-0.23, 0.18]		
Motivation	-0.06	[-0.09, -0.03]			-0.06	[-0.09, -0.03]			-0.07	[-0.09, -0.03]		
Weekend	0.15	[0.11, 0.20]			0.15	[0.11, 0.19]			0.15	[0.11, 0.20]		
SE	-0.34	[-0.55, -0.12]			-0.34	[-0.56, -0.12]			-0.34	[-0.57, -0.13]		
WSE	-0.30	[-0.53, -0.08]			-0.31	[-0.54, -0.08]			-0.31	[-0.54, -0.08]		
Occasion	0.50	[0.39, 0.61]	0.37	[0.31, 0.43]	0.50	[0.40, 0.61]	0.37	[0.31, 0.43]	0.50	[0.40, 0.61]	0.37	[0.31, 0.43]
Time	0.03	[-0.03, 0.10]	0.19	[0.16, 0.22]	0.03	[-0.03, 0.09]	0.19	[0.16, 0.22]	0.03	[-0.03, 0.09]	0.19	[0.16, 0.22]
SE: Occasion	-0.18	[-0.34, -0.03]			-0.19	[-0.34, -0.03]			-0.19	[-0.34, -0.03]		
WSE: Occasion	-0.34	[-0.50, -0.18]			-0.34	[-0.50, -0.18]			-0.34	[-0.50, -0.18]		
SE:Time	0.05	[-0.04, 0.14]			0.05	[-0.04, 0.14]			0.05	[-0.04, 0.14]		
WSE:Time	-0.03	[-0.13, 0.06]			-0.03	[-0.13, 0.06]			-0.04	[-0.13, 0.06]		
Occasion:Time	-0.05	[-0.11, 0.02]			-0.05	[-0.12, 0.02]			-0.05	[-0.12, 0.02]		
SE:Occasion:Time	-0.05	[-0.15, 0.05]			-0.05	[-0.14, 0.05]			-0.04	[-0.14, 0.05]		
WSE:Occasion:Time	0.04	[-0.07, 0.14]			0.04	[-0.07, 0.14]			0.04	[-0.07, 0.14]		

Note. *b* = mean regression coefficient across persons; SD_{Id} = standard deviation of the regression coefficient across persons; 95%-CI = 95%-credibility interval. SE/WSE = (waitlist) socio-emotional training, MB = mindfulness-based training. CoT = Cube of Thought, AG = Affect Grid. Models limited to high compliance (at least 80%) and low count of aborts (at most two) used weakly informative priors.

[a] Since ordinal models have multiple intercepts, we do not report them for brevity.

References

[1] Faul, F., Erdfelder, E., Lang, A.-G. & Buchner, A. G* Power 3: A flexible statistical power analysis program for the social, behavioral, and biomedical sciences. *Behav. Res. Methods* **39**, 175–191 (2007).

[2] Kok, B. E. & Singer, T. Effects of Contemplative Dyads on Engagement and Perceived Social Connectedness Over 9 Months of Mental Training: A Randomized Clinical Trial. *JAMA Psychiatry* **74**, 126 (2017).

[3] Singer, T. *et al. The ReSource Project: Background, design, samples, and measurements*. (Max Planck Institute for Human Cognitive and Brain Sciences, 2016).

[4] Liddell, T. M. & Kruschke, J. K. Analyzing ordinal data with metric models: What could possibly go wrong? *J. Exp. Soc. Psychol.* **79**, 328–348 (2018).

[5] Bürkner, P.-C. Advanced Bayesian Multilevel Modeling with the R Package brms. (2017) doi:10.48550/ARXIV.1705.11123.

[6] Bürkner, P.-C. & Vuorre, M. Ordinal Regression Models in Psychology: A Tutorial. *Adv. Methods Pract. Psychol. Sci.* **2**, 77–101 (2019).

[7] Bürkner, P.-C. **brms** : An *R* Package for Bayesian Multilevel Models Using *Stan*. *J. Stat. Softw.* **80**, (2017).

[8] Vehtari, A., Gelman, A. & Gabry, J. Practical Bayesian model evaluation using leave-one-out cross-validation and WAIC. *Stat. Comput.* **27**, 1413–1432 (2017).
